# 2-Amino­benzoxazole–oxalic acid (2/1)

**DOI:** 10.1107/S2414314624000336

**Published:** 2024-01-26

**Authors:** Surayyo Razzoqova, Batirbay Torambetov, Jamoliddin Todjiev, Shakhnoza Kadirova, Aziz Ibragimov, Abror Ruzmetov, Jamshid Ashurov

**Affiliations:** a National University of Uzbekistan named after Mirzo Ulugbek, 4 University St, Tashkent, 100174, Uzbekistan; b Institute of General and Inorganic Chemistry, Academy of Sciences of Uzbekistan, 100170, M. Ulugbek Str 77a, Tashkent, Uzbekistan; c Institute of Bioorganic Chemistry, Academy of Sciences of Uzbekistan, M. Ulugbek, Str, 83, Tashkent, 100125, Uzbekistan; Howard University, USA

**Keywords:** crystal structure, 2-aminobenzoxazole, mol­ecular structure, co-crystal, hydrogen bonds

## Abstract

In the title compound, 2C_7_H_7_N_2_O^+^·C_2_O_4_^2−^, proton transfer from oxalic acid to the N atom of the heterocycle has occurred to form a 2:1 molecular salt. In the extended structure, N—H⋯O hydrogen bonds link the components into [100] chains, which feature *R* ^2^_2_(8) and *R* ^4^_4_(14) loops.

## Abstract 2

In the title compound, proton transfer from oxalic acid to the N atom of the heterocycle has occurred to form a 2:1 molecular salt. In the extended structure, N—H⋯O hydrogen bonds link the components into [100] chains, which feature *R* ^2^_2_(8) and *R* ^4^_4_(14) loops.

## Graphical abstract

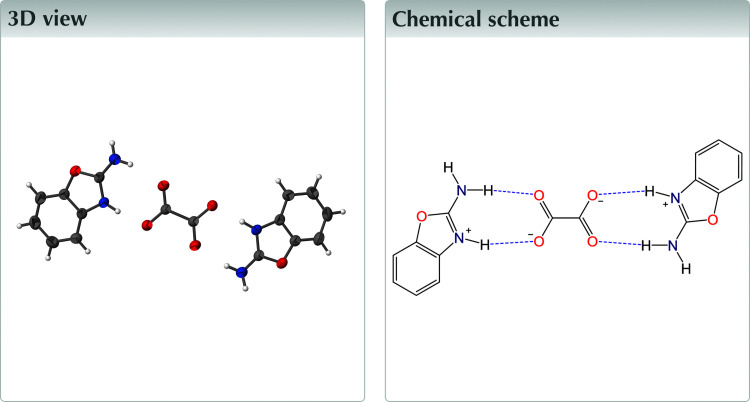

## Structure description

2-Amino­benzoxazole has gained significant attention in the field of organic chemistry due to its diverse range of applications and properties. This heterocyclic compound exhibits intriguing structural features and has demonstrated potential utility in the development of pharmaceuticals and agrochemicals and in materials science (Hwang *et al.*, 2006 ▸; Potashman *et al.*, 2007 ▸). With its aromatic and nitro­gen-containing structural motifs, 2-amino­benzoxazole has emerged as a key scaffold for the synthesis of biologically active mol­ecules and advanced materials. Herein, we report on the crystal structure analysis of a new 2-amino­benzoxazole–oxalic acid mol­ecular salt.

**Figure 1:**
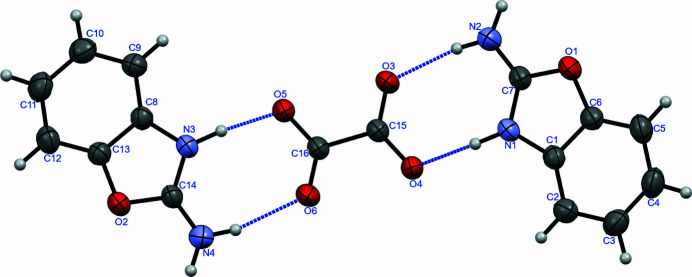
The organic salt structure of 2ABO and OXL. Displacement ellipsoids are drawn at the 50% probability level and N—H⋯O hydrogen bonds are shown as dashed lines.

**Figure 2:**
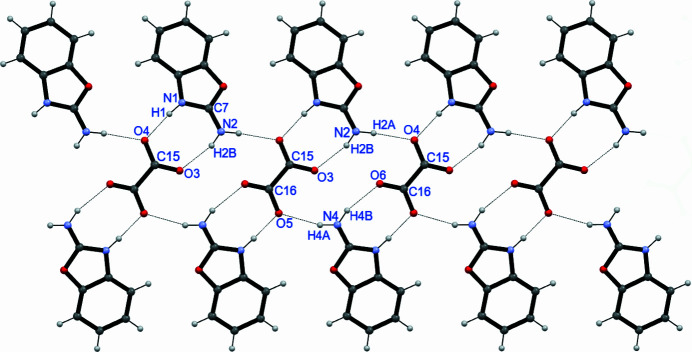
The crystal structure of the organic salt structure of 2ABO and OXL viewed along the *c* axis.

**Figure 3:**
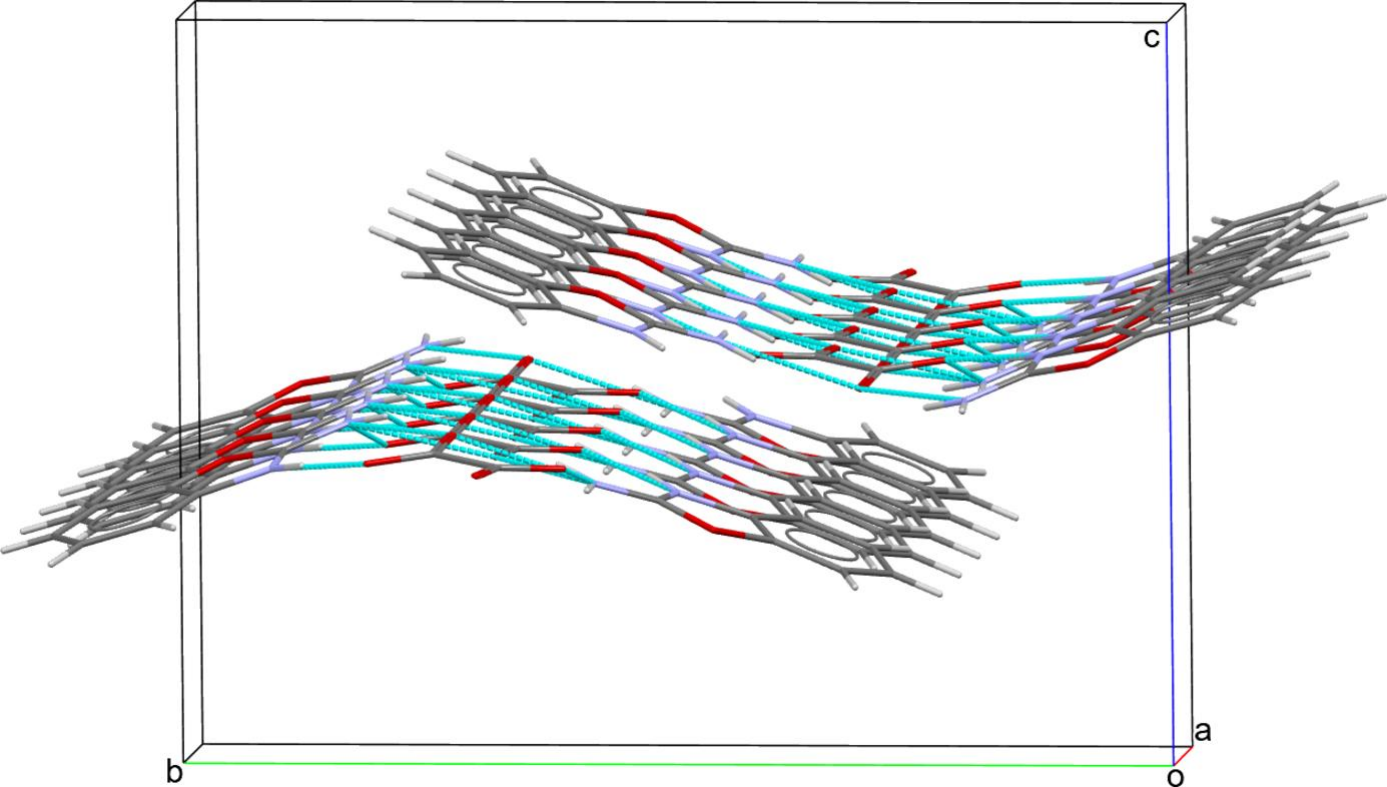
A fragment of a [100] chain in the extended structure of the title compound with hydrogen bonds shown as dashed lines.

The title organic salt crystallizes in the monoclinic space group *P*2_1_/*n*. The mol­ecular structure of the organic salt is shown in Fig. 1 ▸. The geometric parameters of the arene and oxazole rings are similar to standard values and to those in other related structures (Ashurov *et al.*, 2011 ▸, 2015 ▸; Wang *et al.*, 2016 ▸). In the oxalate (OXL) part of the organic salt, two hydrogen atoms are transferred to the nitro­gen of the oxazole fragments, as in other 2-amino­benzoxazole (2ABO) structures (Nandy *et al.*, 2016 ▸; Razzoqova *et al.*, 2022 ▸, 2023 ▸). As a result, the 2ABO and OXL ions form two closed eight-membered rings with an 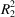 (8) graph-set notation (Etter *et al.*, 1990 ▸). This represents a 1:2 acid-base association (Calva *et al.*, 2011 ▸), with the first ring formed by N1—H1⋯O4 and N2—H2*B*⋯O3 hydrogen bonds and the second by N4—H4*B*⋯O6 and N3—H3*A*⋯O5 hydrogen bonds (Table 1 ▸). N2—H2*A*⋯O4 and N4—H4*A*⋯O5 hydrogen bonds further link the components into [100] chains, thereby forming a 14-membered ring with an 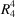 (14) graph-set motif (Fig. 2 ▸) (Etter *et al.*, 1990 ▸). The chains are shown in Fig. 3 ▸.

The identification of the co-crystal as a salt is based on the successful refinement of the relevant H atoms using X-ray data. The proton transfer is further supported by the C—O distances [O4—C15 = 1.266 (2) Å, O3—C15 = 1.234 (2) Å, O5—C16 = 1.272 (2) Å and O6—C16 = 1.222 (2) Å] with differences between the bond lengths within each group of 0.032 and 0.050 Å; these differences differ from those for O—C distances in deproton­ated carboxyl groups. In non-deprotonated oxalic acid, these differences are greater (Sasaki *et al.*, 2020 ▸). The mean planes of the carb­oxy­lic fragments in the OXL ion are turned by 10.13 (4)° from each other. In the crystal, the 2BAO and OXL ions are not coplanar, the 2ABO ions being inclined to the OXL ions by 18.81 (3) and 16.00 (5)°. The dihedral angle between the 2ABO ions is 37.52 (2)°.

## Synthesis and crystallization

A 2:1 stoichiometric ratio of 2-amino­benzoxazole (0.268 g, 2.0 mmol) and oxalic acid (0.090 g, 1.0 mmol) was dissolved and mixed well in distilled water (5 ml). The mixture was stirred at room temperature for 30 minutes. The solution was then transferred to a vial with small holes in the cover to allow for evaporation. After about 3 weeks, cube-like single crystals of the title salt suitable for data collection were obtained.

## Refinement

Crystal data, data collection and structure refinement details are summarized in Table 2 ▸.

## Supplementary Material

Crystal structure: contains datablock(s) I. DOI: 10.1107/S2414314624000336/bv4051sup1.cif

Supporting information file. DOI: 10.1107/S2414314624000336/bv4051Isup2.cml

CCDC reference: 2324364

Additional supporting information:  crystallographic information; 3D view; checkCIF report

## Figures and Tables

**Table 1:** Hydrogen-bond geometry (Å, °)

*D*—H⋯*A*	*D*—H	H⋯*A*	*D*⋯*A*	*D*—H⋯*A*
N1—H1⋯O4	0.88 (1)	1.78 (1)	2.650 (2)	176 (3)
N2—H2*A*⋯O4^i^	0.87 (1)	2.00 (1)	2.863 (2)	174 (3)
N2—H2*B*⋯O3	0.86 (1)	1.94 (1)	2.770 (2)	164 (2)
C2—H2⋯O1^ii^	0.93	2.65	3.522 (2)	157
N3—H3*A*⋯O5	0.88 (1)	1.71 (1)	2.577 (2)	173 (3)
N4—H4*A*⋯O5^ii^	0.87 (1)	2.06 (1)	2.918 (2)	172 (2)
N4—H4*B*⋯O6	0.86 (1)	2.02 (1)	2.851 (2)	163 (2)

Symmetry codes: (i) 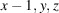 ; (ii) 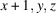 .

**Table 2:** Experimental details

Crystal data	
Chemical formula	2C_7_H_7_N_2_O^+^·C_2_O_4_^2−^
*M*_r_	358.31
Crystal system, space group	Monoclinic, *P*2_1_/*n*
Temperature (K)	293
*a*, *b*, *c* (Å)	6.5080 (2), 17.6943 (7), 13.6264 (5)
β (°)	100.200 (4)
*V* (Å^3^)	1544.34 (10)
*Z*	4
Radiation type	Cu *K*α
μ (mm^−1^)	1.03
Crystal size (mm)	0.17 × 0.14 × 0.12

Data collection	
Diffractometer	XtaLAB Synergy, Single source at home/near, HyPix3000
Absorption correction	Multi-scan (*CrysAlis PRO*; Rigaku OD, 2020 ▸)
*T*_min_, *T*_max_	0.157, 1.000
No. of measured, independent and observed [*I* > 2σ(*I*)] reflections	2960, 2960, 2288
*R*_int_	0.031
(sin θ/λ)_max_ (Å^−1^)	0.615

Refinement	
*R*[*F* ^2^ > 2σ(*F* ^2^)], *wR*(*F* ^2^), *S*	0.045, 0.132, 1.04
No. of reflections	2960
No. of parameters	259
No. of restraints	6
H-atom treatment	H atoms treated by a mixture of independent and constrained refinement
Δρ_max_, Δρ_min_ (e Å^−3^)	0.23, −0.20

Computer programs: *CrysAlis PRO* (Rigaku OD, 2020 ▸), *SHELXT2018/2* (Sheldrick, 2015*a* ▸), *SHELXL2019/3* (Sheldrick, 2015*b* ▸) and *OLEX2* (Dolomanov *et al.*, 2009 ▸).

## full crystallographic data

### Crystal data

**Table:**

2C_7_H_7_N_2_O^+^·C_2_O_4_^2^^−^	*F*(000) = 744
*M**_r_* = 358.31	*D*_x_ = 1.541 Mg m^−^^3^
Monoclinic, *P*2_1_/*n*	Cu *K*α radiation, λ = 1.54184 Å
*a* = 6.5080 (2) Å	Cell parameters from 2449 reflections
*b* = 17.6943 (7) Å	θ = 4.1–70.3°
*c* = 13.6264 (5) Å	µ = 1.03 mm^−^^1^
β = 100.200 (4)°	*T* = 293 K
*V* = 1544.34 (10) Å^3^	Needle, light yellow
*Z* = 4	0.17 × 0.14 × 0.12 mm

### Data collection

**Table:**

XtaLAB Synergy, Single source at home/near, HyPix3000 diffractometer	2960 independent reflections
Radiation source: micro-focus sealed X-ray tube, PhotonJet (Cu) X-ray Source	2288 reflections with *I* > 2σ(*I*)
Mirror monochromator	*R*_int_ = 0.031
Detector resolution: 10.0000 pixels mm^-1^	θ_max_ = 71.4°, θ_min_ = 4.1°
ω scans	*h* = −8→7
Absorption correction: multi-scan (CrysAlisPro; Rigaku OD, 2020)	*k* = −20→21
*T*_min_ = 0.157, *T*_max_ = 1.000	*l* = −13→16
2960 measured reflections	

### Refinement

**Table:**

Refinement on *F*^2^	Primary atom site location: dual
Least-squares matrix: full	Hydrogen site location: mixed
*R*[*F*^2^ > 2σ(*F*^2^)] = 0.045	H atoms treated by a mixture of independent and constrained refinement
*wR*(*F*^2^) = 0.132	*w* = 1/[σ^2^(*F*_o_^2^) + (0.0724*P*)^2^ + 0.1538*P*] where *P* = (*F*_o_^2^ + 2*F*_c_^2^)/3
*S* = 1.03	(Δ/σ)_max_ < 0.001
2960 reflections	Δρ_max_ = 0.23 e Å^−^^3^
259 parameters	Δρ_min_ = −0.20 e Å^−^^3^
6 restraints	

### Special details

**Table:**

Geometry. All esds (except the esd in the dihedral angle between two l.s. planes) are estimated using the full covariance matrix. The cell esds are taken into account individually in the estimation of esds in distances, angles and torsion angles; correlations between esds in cell parameters are only used when they are defined by crystal symmetry. An approximate (isotropic) treatment of cell esds is used for estimating esds involving l.s. planes.
Refinement. The hydrogen atoms of amino groups and protonated nitro­gen atoms of oxzole groups were located in difference - Fourier maps and refined with restrained distances of 0.85±(1) Å. The H atoms of the benzene ring were calculated geometrically with C—H = 0.93 Å and *U*_iso_(H) = 1.2*U*_eq_(C).

### Fractional atomic coordinates and isotropic or equivalent isotropic displacement parameters (Å^2^)

**Table:**

	*x*	*y*	*z*	*U*_iso_*/*U*_eq_	
O1	0.1212 (2)	0.44529 (8)	0.33805 (10)	0.0428 (3)	
N1	0.4453 (2)	0.48755 (9)	0.38019 (11)	0.0381 (4)	
H1	0.545 (3)	0.5203 (13)	0.3995 (19)	0.082 (9)*	
C1	0.4597 (3)	0.41299 (11)	0.34764 (13)	0.0366 (4)	
O2	1.3397 (2)	0.94256 (8)	0.42539 (10)	0.0410 (3)	
N2	0.1639 (3)	0.56637 (11)	0.39934 (16)	0.0521 (5)	
H2A	0.0351 (19)	0.5701 (15)	0.4069 (18)	0.069 (8)*	
H2B	0.252 (3)	0.6021 (11)	0.4168 (18)	0.069 (8)*	
C2	0.6255 (3)	0.36637 (12)	0.34088 (15)	0.0439 (5)	
H2	0.762934	0.382283	0.360114	0.053*	
O3	0.4839 (2)	0.67161 (8)	0.42640 (12)	0.0540 (4)	
N3	1.0284 (2)	0.88951 (9)	0.42134 (12)	0.0381 (4)	
H3A	0.927 (3)	0.8578 (14)	0.427 (2)	0.090 (10)*	
C3	0.5769 (4)	0.29442 (12)	0.30389 (16)	0.0518 (5)	
H3	0.685094	0.261048	0.299092	0.062*	
O4	0.7441 (2)	0.58949 (7)	0.42948 (10)	0.0432 (3)	
N4	1.3345 (3)	0.81860 (10)	0.47545 (14)	0.0470 (4)	
H4A	1.4629 (19)	0.8123 (15)	0.4680 (17)	0.061 (7)*	
H4B	1.258 (3)	0.7797 (10)	0.4805 (18)	0.064 (8)*	
C4	0.3719 (4)	0.27030 (13)	0.27364 (17)	0.0533 (6)	
H4	0.346243	0.222044	0.247253	0.064*	
O5	0.7522 (2)	0.78759 (8)	0.43539 (12)	0.0517 (4)	
C5	0.2049 (3)	0.31731 (12)	0.28231 (16)	0.0497 (5)	
H5	0.066961	0.301904	0.263314	0.060*	
O6	1.0164 (2)	0.70685 (9)	0.46759 (14)	0.0624 (5)	
C6	0.2561 (3)	0.38746 (11)	0.32045 (13)	0.0394 (4)	
C7	0.2444 (3)	0.50366 (11)	0.37490 (13)	0.0383 (4)	
C8	0.9941 (3)	0.96341 (10)	0.38748 (13)	0.0350 (4)	
C9	0.8136 (3)	1.00364 (12)	0.35338 (13)	0.0420 (4)	
H9	0.682079	0.982192	0.350086	0.050*	
C10	0.8389 (3)	1.07772 (13)	0.32431 (15)	0.0490 (5)	
H10	0.720945	1.106402	0.300336	0.059*	
C11	1.0345 (4)	1.11046 (13)	0.32983 (16)	0.0531 (5)	
H11	1.044476	1.160508	0.310364	0.064*	
C12	1.2147 (3)	1.06995 (12)	0.36377 (16)	0.0483 (5)	
H12	1.346707	1.091225	0.368116	0.058*	
C13	1.1870 (3)	0.99662 (11)	0.39057 (13)	0.0377 (4)	
C14	1.2326 (3)	0.87935 (11)	0.44171 (13)	0.0372 (4)	
C15	0.6703 (3)	0.65540 (10)	0.43310 (13)	0.0354 (4)	
C16	0.8301 (3)	0.72137 (11)	0.44698 (14)	0.0379 (4)	

### Atomic displacement parameters (Å^2^)

**Table:**

	*U*^11^	*U*^22^	*U*^33^	*U*^12^	*U*^13^	*U*^23^
O1	0.0305 (7)	0.0392 (7)	0.0574 (8)	−0.0051 (5)	0.0037 (5)	−0.0028 (6)
N1	0.0284 (8)	0.0363 (9)	0.0492 (8)	−0.0048 (6)	0.0058 (6)	−0.0030 (7)
C1	0.0356 (10)	0.0348 (10)	0.0396 (9)	−0.0046 (8)	0.0071 (7)	0.0037 (7)
O2	0.0322 (7)	0.0382 (7)	0.0536 (8)	−0.0047 (5)	0.0098 (5)	0.0007 (6)
N2	0.0331 (9)	0.0404 (10)	0.0844 (13)	−0.0023 (8)	0.0144 (9)	−0.0085 (9)
C2	0.0377 (11)	0.0428 (11)	0.0523 (11)	0.0007 (8)	0.0110 (8)	0.0039 (9)
O3	0.0292 (7)	0.0391 (8)	0.0945 (11)	−0.0031 (6)	0.0131 (7)	−0.0047 (8)
N3	0.0298 (8)	0.0342 (9)	0.0515 (9)	−0.0049 (7)	0.0101 (6)	−0.0018 (7)
C3	0.0567 (14)	0.0396 (11)	0.0626 (13)	0.0058 (10)	0.0202 (10)	0.0046 (10)
O4	0.0327 (7)	0.0323 (7)	0.0645 (8)	−0.0021 (5)	0.0086 (6)	−0.0027 (6)
N4	0.0360 (10)	0.0392 (10)	0.0663 (11)	0.0014 (8)	0.0102 (8)	0.0037 (8)
C4	0.0648 (15)	0.0344 (11)	0.0637 (13)	−0.0072 (10)	0.0199 (11)	−0.0045 (10)
O5	0.0324 (7)	0.0332 (7)	0.0900 (11)	−0.0013 (6)	0.0120 (7)	0.0054 (7)
C5	0.0486 (12)	0.0430 (12)	0.0579 (12)	−0.0135 (9)	0.0105 (9)	−0.0027 (9)
O6	0.0291 (8)	0.0397 (9)	0.1155 (14)	−0.0015 (6)	0.0047 (8)	0.0032 (8)
C6	0.0351 (10)	0.0378 (10)	0.0451 (10)	−0.0019 (8)	0.0063 (7)	0.0037 (8)
C7	0.0328 (9)	0.0344 (10)	0.0477 (10)	−0.0039 (7)	0.0066 (7)	0.0009 (8)
C8	0.0355 (10)	0.0337 (10)	0.0367 (8)	−0.0029 (7)	0.0093 (7)	−0.0032 (7)
C9	0.0353 (10)	0.0433 (11)	0.0476 (10)	0.0004 (8)	0.0082 (8)	−0.0014 (9)
C10	0.0491 (12)	0.0459 (12)	0.0525 (11)	0.0074 (9)	0.0102 (9)	0.0043 (9)
C11	0.0620 (14)	0.0383 (12)	0.0616 (13)	−0.0022 (10)	0.0180 (10)	0.0085 (10)
C12	0.0476 (12)	0.0418 (12)	0.0576 (12)	−0.0100 (9)	0.0154 (9)	0.0013 (9)
C13	0.0361 (10)	0.0357 (10)	0.0423 (9)	−0.0014 (8)	0.0098 (7)	−0.0029 (8)
C14	0.0330 (10)	0.0340 (10)	0.0453 (9)	−0.0050 (7)	0.0090 (7)	−0.0041 (8)
C15	0.0304 (9)	0.0343 (10)	0.0420 (9)	−0.0025 (7)	0.0079 (7)	0.0009 (8)
C16	0.0305 (9)	0.0336 (10)	0.0498 (10)	−0.0033 (7)	0.0077 (7)	0.0007 (8)

### Geometric parameters (Å, º)

**Table:**

O1—C6	1.397 (2)	N4—H4A	0.866 (10)
O1—C7	1.349 (2)	N4—H4B	0.858 (10)
N1—H1	0.876 (10)	N4—C14	1.303 (3)
N1—C1	1.400 (3)	C4—H4	0.9300
N1—C7	1.328 (2)	C4—C5	1.390 (3)
C1—C2	1.374 (3)	O5—C16	1.276 (2)
C1—C6	1.386 (3)	C5—H5	0.9300
O2—C13	1.400 (2)	C5—C6	1.364 (3)
O2—C14	1.357 (2)	O6—C16	1.222 (2)
N2—H2A	0.865 (10)	C8—C9	1.382 (3)
N2—H2B	0.857 (10)	C8—C13	1.380 (3)
N2—C7	1.296 (3)	C9—H9	0.9300
C2—H2	0.9300	C9—C10	1.388 (3)
C2—C3	1.385 (3)	C10—H10	0.9300
O3—C15	1.234 (2)	C10—C11	1.388 (3)
N3—H3A	0.877 (10)	C11—H11	0.9300
N3—C8	1.391 (2)	C11—C12	1.382 (3)
N3—C14	1.321 (2)	C12—H12	0.9300
C3—H3	0.9300	C12—C13	1.369 (3)
C3—C4	1.392 (3)	C15—C16	1.553 (3)
O4—C15	1.266 (2)		
			
C7—O1—C6	105.93 (14)	C5—C6—C1	123.77 (19)
C1—N1—H1	129 (2)	N1—C7—O1	111.77 (16)
C7—N1—H1	123 (2)	N2—C7—O1	120.69 (17)
C7—N1—C1	107.77 (15)	N2—C7—N1	127.53 (18)
C2—C1—N1	133.15 (18)	C9—C8—N3	132.26 (18)
C2—C1—C6	120.73 (18)	C13—C8—N3	107.36 (16)
C6—C1—N1	106.12 (16)	C13—C8—C9	120.37 (18)
C14—O2—C13	105.31 (14)	C8—C9—H9	121.8
H2A—N2—H2B	122 (3)	C8—C9—C10	116.46 (19)
C7—N2—H2A	122.7 (18)	C10—C9—H9	121.8
C7—N2—H2B	115.0 (19)	C9—C10—H10	118.9
C1—C2—H2	121.8	C9—C10—C11	122.2 (2)
C1—C2—C3	116.40 (19)	C11—C10—H10	118.9
C3—C2—H2	121.8	C10—C11—H11	119.4
C8—N3—H3A	123 (2)	C12—C11—C10	121.1 (2)
C14—N3—H3A	129 (2)	C12—C11—H11	119.4
C14—N3—C8	107.10 (15)	C11—C12—H12	122.1
C2—C3—H3	118.8	C13—C12—C11	115.9 (2)
C2—C3—C4	122.3 (2)	C13—C12—H12	122.1
C4—C3—H3	118.8	C8—C13—O2	107.87 (16)
H4A—N4—H4B	119 (2)	C12—C13—O2	128.21 (18)
C14—N4—H4A	120.4 (17)	C12—C13—C8	123.92 (19)
C14—N4—H4B	115.1 (18)	N3—C14—O2	112.34 (16)
C3—C4—H4	119.5	N4—C14—O2	119.57 (17)
C5—C4—C3	120.9 (2)	N4—C14—N3	128.08 (18)
C5—C4—H4	119.5	O3—C15—O4	125.89 (17)
C4—C5—H5	122.1	O3—C15—C16	117.56 (16)
C6—C5—C4	115.8 (2)	O4—C15—C16	116.55 (16)
C6—C5—H5	122.1	O5—C16—C15	115.62 (16)
C1—C6—O1	108.39 (16)	O6—C16—O5	125.33 (18)
C5—C6—O1	127.84 (18)	O6—C16—C15	119.06 (17)
			
N1—C1—C2—C3	−179.9 (2)	C6—C1—C2—C3	1.4 (3)
N1—C1—C6—O1	−1.0 (2)	C7—O1—C6—C1	0.03 (19)
N1—C1—C6—C5	178.36 (18)	C7—O1—C6—C5	−179.3 (2)
C1—N1—C7—O1	−1.8 (2)	C7—N1—C1—C2	−177.2 (2)
C1—N1—C7—N2	179.0 (2)	C7—N1—C1—C6	1.7 (2)
C1—C2—C3—C4	0.9 (3)	C8—N3—C14—O2	1.1 (2)
C2—C1—C6—O1	178.00 (16)	C8—N3—C14—N4	179.93 (19)
C2—C1—C6—C5	−2.6 (3)	C8—C9—C10—C11	−0.6 (3)
C2—C3—C4—C5	−2.0 (3)	C9—C8—C13—O2	−178.45 (16)
O3—C15—C16—O5	−10.1 (3)	C9—C8—C13—C12	1.8 (3)
O3—C15—C16—O6	169.97 (19)	C9—C10—C11—C12	0.8 (3)
N3—C8—C9—C10	−179.46 (19)	C10—C11—C12—C13	0.3 (3)
N3—C8—C13—O2	0.65 (19)	C11—C12—C13—O2	178.73 (18)
N3—C8—C13—C12	−179.10 (18)	C11—C12—C13—C8	−1.6 (3)
C3—C4—C5—C6	0.9 (3)	C13—O2—C14—N3	−0.71 (19)
O4—C15—C16—O5	169.79 (17)	C13—O2—C14—N4	−179.63 (17)
O4—C15—C16—O6	−10.2 (3)	C13—C8—C9—C10	−0.6 (3)
C4—C5—C6—O1	−179.32 (18)	C14—O2—C13—C8	0.00 (18)
C4—C5—C6—C1	1.4 (3)	C14—O2—C13—C12	179.74 (19)
C6—O1—C7—N1	1.1 (2)	C14—N3—C8—C9	177.88 (19)
C6—O1—C7—N2	−179.66 (18)	C14—N3—C8—C13	−1.07 (19)

### Hydrogen-bond geometry (Å, º)

**Table:**

*D*—H···*A*	*D*—H	H···*A*	*D*···*A*	*D*—H···*A*
N1—H1···O4	0.88 (1)	1.78 (1)	2.650 (2)	176 (3)
N2—H2*A*···O4^i^	0.87 (1)	2.00 (1)	2.863 (2)	174 (3)
N2—H2*B*···O3	0.86 (1)	1.94 (1)	2.770 (2)	164 (2)
C2—H2···O1^ii^	0.93	2.65	3.522 (2)	157
N3—H3*A*···O5	0.88 (1)	1.71 (1)	2.577 (2)	173 (3)
N4—H4*A*···O5^ii^	0.87 (1)	2.06 (1)	2.918 (2)	172 (2)
N4—H4*B*···O6	0.86 (1)	2.02 (1)	2.851 (2)	163 (2)

Symmetry codes: (i) *x*−1, *y*, *z*; (ii) *x*+1, *y*, *z*.
